# Comment on “Comparing open conventional carpal tunnel release with mini-incision technique in the treatment of carpal tunnel syndrome - a non-randomized clinical trial”

**DOI:** 10.1016/j.amsu.2020.07.048

**Published:** 2020-08-12

**Authors:** Alex Haiser, Chloe L. Jordan

**Affiliations:** a Barts and The London School of Medicine and Dentistry, Queen Mary University of London, London, United Kingdom; b Imperial College School of Medicine, Imperial College London, London, United Kingdom

To the editor:

We read with great interest the article “*Comparing open conventional carpal tunnel release with mini-incision technique in the treatment of carpal tunnel syndrome-a non-randomized clinical trial”* [1] by Khoshnevis et al.

Carpal tunnel syndrome (CTS) is the most common entrapment mononeuropathy worldwide, accounting for 90% of all cases [2]. With surgical management becoming increasingly common [3], there is a debate as to whether minimally invasive carpal tunnel release (MICTR) is superior to conventional open carpal tunnel release (OCTR). Recent literature has highlighted this controversy, however there is currently a lack of evidence to influence a shift in practice and identify the superiority of MICTR. Currently, the choice of which technique to perform depends on the preference of both surgeon and patient [[4], [5], [6]]. We congratulate the authors for addressing the highly relevant need to identify the superior technique in order to improve patient outcomes.

This study was well-conducted and there are undoubtedly many strengths, some of which will be discussed. However, there are also some suggestions, which we believe would benefit this research with regards to the impact on clinical practice.

We praise the author's comprehensive documentation of the methodology, which covered a clear and concise description of both surgical procedures and the expert surgeons who performed them. Furthermore, researchers ensured that all measures were the same for all participants with the only difference being the type of surgery, allowing for a causal relationship to be explored. The inclusion and exclusion criteria were suitable due to the use of both qualitative techniques; Phalen's and Tinel's and quantitative tests; Electromyography and Nerve Conduction Velocity (EMG-NCV), thereby creating a suitable patient cohort [1].

However, the trial was only conducted at a single centre in Iran and involved a relatively small sample of patients. This is a major limitation of this study as a multi-centre study with a greater patient population is required to validate these results and to be more representative of the global population. Furthermore, patients with diabetes mellitus and thyroid disorders were excluded from the study population [1]. Various studies have demonstrated that both conditions have a negative impact on post-CTR outcomes [7,8]. This raises the question that if these patients were included in the trial, this may have led to different results. Including a well-balanced population, with patients who are at risk of increased post-operative complications, is needed to ensure a fair trial.

Upon examining the patient population, it is evident that not only was there an unequal number of patients in each group, but the sample was also predominantly female [1]. Despite the gender disparities, literature has highlighted that CTS is more prevalent in females and thus, the study is realistic in simulating a patient population [2]. Finally, and most importantly, the authors failed to randomise the study population. Randomisation is an important aspect in any clinical trial to ensure that selection bias is not present. Therefore, randomisation in this trial is crucial in order to make the results more reliable. Nevertheless, we give credit to the authors who have acknowledged this point as a potential trial limitation.

Establishing clear and well-defined outcomes that evaluate both short and long-term patient outcomes is paramount in order to effectively compare surgical techniques. CTS is a chronic disease affecting patients throughout their lifetime, but there are current shortcomings to the long-term outcome literature. Upon review of the literature, CTS recurrence rates range from 3% to 25%. Studies have reported that resolution of paraesthesia may not occur until a minimum of 9 months following surgery [9]. Concannon et al. reported a statistically higher incidence of recurrence of CTS after endoscopic release compared with the traditional “open.” [10].

Recurrence is an important outcome that authors have failed to include within this study, as recurrence affects patient satisfaction, health related quality of life and physical functioning. This study period, 12 months, may not be sufficient to capture the full range of clinical outcomes and therefore conclusions cannot be drawn on the superiority of either technique. To further progress this work, patients should be followed up for a minimum of 24 months. A longer follow-up period would enable CTS recurrence to be recorded for both techniques and also to evaluate other long-term outcomes.

Eligibility for surgery is determined on the severity of CTS, yet the authors do not mention the EMG-NCV applied to categorise CTS severity. Studies that have used EMG/NCV have confirmed that although distal latency scores have improved following surgery, in the majority of cases they do not return to normal range [11](9). Abnormal latencies can be prolonged with the literature reporting elevated scores for a duration greater than 36 months, once again highlighting the importance of a longitudinal study in order to adequately assess long-term outcomes between both MICTR and OCTR [12,13].

Authors assessed postoperative pain using visual analogue scale (VAS) scores. The VAS is a validated measure for pain, with the scale being widely and successfully used in the literature [14]. Authors used VAS scores for pain at rest and during activity, however a study by Meirelles et al. reported that nocturnal paraesthesia was the most optimal marker of improvement following carpal tunnel release surgery [15]. Many studies which have compared treatment modalities for CTS have included nocturnal paraesthesia as one of their outcomes, one of which the authors of this paper have failed to include and therefore could consider for future works [[16], [17], [18]].

In summary, we believe that much of the study design is strong but would benefit from the aforementioned. A longitudinal, multicentre trial with randomisation of participants would produce more valid and clinically applicable results. Thus, future studies which take into account our suggestions could contribute to a change in guidelines for the surgical management for CTS, consequently improving patient outcomes.

## Ethics

No IRB approval or consent was required for this work.

## Sources of funding

N/A.

## Author contribution

Both authors (Chloe Jordan and Alex Haiser) have contributed significantly and equally to this letter to the editor. The structure and the writing of the letter was completed by both authors who collaboratively produced this piece of work.

## Trials register

Name of the registry: N/A.

Unique Identifying number or registration ID: N/A.

Hyperlink to your specific registration (must be publicly accessible and will be checked): N/A.

## Guarantor

N/A.

## Consent

N/A.

## Provenance and peer review

Not commissioned, editor reviewed.

## Declaration of competing interest

Nothing to declare.
